# Dietary Supplementation with Yak Stomach Lysozyme Improves Intestinal Health and Nutrient Metabolism in Weaned Piglets Challenged with Enterotoxigenic *Escherichia coli* (ETEC)

**DOI:** 10.3390/ani15223287

**Published:** 2025-11-13

**Authors:** Zaiwen Li, Lian Hu, Mengjuan Jiang, Di Zhao, Lu Yang, Yili Liu, Biao Li, Mingfeng Jiang

**Affiliations:** 1 Key Laboratory of Qinghai-Tibetan Plateau Animal Genetic Resource Reservation and Utilization, Ministry of Education and Sichuan Province, Southwest Minzu University, Chengdu 610041, China; 18308211268@163.com (Z.L.); 18188486427@163.com (L.H.); 15037052213@163.com (M.J.); 80300163@swun.edu.cn (Y.L.); 2 College of Animal Science and Veterinary Medicine, Southwest Minzu University, Chengdu 610041, China

**Keywords:** yak stomach lysozyme, piglet diarrhea, proteomics, metabolomics

## Abstract

Post-weaning diarrhea caused by Enterotoxigenic *Escherichia coli* (ETEC) is a major disease in piglets and leads to substantial economic losses in the swine industry. Compared to conventional lysozyme, yak stomach lysozyme (YSL) demonstrates distinctive resistance to pepsin, trypsin, high temperature, and acidic conditions. This study investigated the effects of dietary YSL supplementation on intestinal health in weaned piglets challenged with ETEC, utilizing metabolomics and proteomics. A total of 18 weaned piglets were randomly divided into three groups: control (C), diarrhea (D), and YSL treatment (YLT). Groups C and D were fed a basal diet, while the YLT group received the basal diet supplemented with YSL at a dosage of 100,000 U/kg following ETEC challenge. Following an acclimation period, piglets in groups D and YLT were orally challenged with ETEC, while group C received the same volume of sterile LB broth. The feeding trial lasted for 21 days before sample collection. The results demonstrated that dietary supplementation with YSL significantly reduced the diarrhea rate (*p* < 0.05). Compared with the D group, the YLT group exhibited significantly increased serum albumin levels (*p* < 0.05), along with a tendency toward greater villus height (*p* = 0.085) and higher serum glucose levels (*p* = 0.052), indicating an improvement in nutritional and metabolic status Metabolomic analysis identified 260 differentially abundant metabolites between the YLT and D groups (81 upregulated, 179 downregulated), which were predominantly enriched in pathways related to amino acid biosynthesis and metabolism, purine metabolism, and nucleic acid metabolism. Proteomic profiling revealed 571 differentially expressed proteins (237 upregulated, 334 downregulated). Upregulated proteins were mainly involved in arginine biosynthesis and base excision repair, while downregulated proteins were associated with the PPAR signaling pathway and Salmonella infection. In summary, dietary YSL supplementation alters the metabolic and proteomic profiles in the intestines of diarrheic piglets, potentially improving gut barrier function and nutrient utilization. This study offers novel insights into the potential of YSL as a promising feed additive for prevention of post-weaning diarrhea in pigs.

## Simple Summary

Enterotoxigenic *Escherichia coli* (ETEC)-induced post-weaning diarrhea severely impairs the health of weaned piglets and inflicts considerable economic losses on the swine industry. Antibiotics were once extensively employed to treat this diarrhea; yet, they have triggered concerns over drug residues and antimicrobial resistance, rendering the development of safe alternatives an urgent necessity. Yak stomach lysozyme (YSL) exhibits unique advantages, including resistance to pepsin, trypsin, high-temperature tolerance, and acid stability—traits that enable it to retain biological activity in the gastrointestinal tract of piglets. In this study, we first induced diarrhea in weaned piglets through ETEC challenge, and then provided the treatment group with a diet supplemented with YSL at a concentration of 100,000 U/kg. At the end of the experiment, the YSL-supplemented group exhibited a significantly lower diarrhea incidence and improved systemic nutritional status. Moreover, YSL was shown to modulate intestinal metabolite profiles and regulate the expression of key intestinal proteins, thereby mitigating intestinal epithelial damage and exerting a therapeutic effect against ETEC-induced diarrhea.

## 1. Introduction

The rearing of weaned piglets is of great significance in pig production. At this stage, the digestive and immune systems of weaned piglets are not fully developed [1]. The drastic changes in the internal and external environments after weaning can easily lead to diarrhea, and ETEC is the main pathogen [2]. The high infection rate and mortality of piglet diarrhea have caused huge economic losses to the global pig farming industry. Although supplementing feed with antibiotics can effectively treat diarrhea [3], the widespread use of these drugs has resulted in residues in animal products and increased antibiotic resistance among livestock, posing significant threats to human health [4]. Many countries have begun to mandate the prohibition of antibiotic use in pig production gradually or are seeking alternatives to antibiotics [5,6].

Lysozyme possesses antibacterial, anti-inflammatory, immunomodulatory and antiviral properties, and it is widely present in nature [7]. Therefore, in recent years, the application of lysozyme has become increasingly extensive [8]. For instance, the European Union has incorporated lysozyme into its list of food additives. The U.S. Food and Drug Administration has classified it as a generally recognized as safe (GRAS) component [9]. In China, lysozyme is considered a conventional additive and is permitted for use in infant formula [10]. In the field of livestock feed, lysozyme can boost the daily weight gain, improve the feed conversion rate of livestock, and enhance animals’ immunity [11,12]. Huang et al. [13] found that neonatal piglets fed with milk from transgenic pigs expressing recombinant human lysozyme exhibited accelerated recovery from ETEC infection, and reduced mortality. Xia et al. [14] found that weaned piglets supplemented with dietary lysozyme showed improved intestinal structure, elevated serum protein levels, and optimized gut microbiota, along with enhanced anti-inflammatory and antioxidant abilities.

Thus, lysozyme has certain application value, with YSL outperforming conventional lysozyme. YSL features resistance to digestive enzymes (pepsin, trypsin), high-temperature tolerance, and acid stability, which enable it to retain activity during high-temperature pelleting, endure the stomach’s acidic and proteolytic environment, and reach the gastrointestinal tract in a functionally intact form. This makes YSL a potential substitute for antibiotics in animal feed. The yak, an economically important bovine endemic to the Tibetan Plateau, produces abomasal secretions that contain exceptionally high levels of lysozyme. A relevant patent early U.S. patent, as well as our research group’s study on this issue and mouse feeding experiments, have also confirmed the above view. In mouse experiment, YSL exhibited a more potent therapeutic effect against ETEC-induced diarrhea in mice than egg white lysozyme and effectively regulated the gut microbiota [15,16]. Additionally, studies have shown that adding lysozyme to the diet can enhance the stress response induced by ETEC in piglets [1]. However, there is no report on the application of YSL as a feed additive in livestock which may be better than egg white lysozyme. Therefore, to further investigate the effects of YSL in major livestock, this study will isolate and purify YSL for use as a feed additive in the diets of weaned piglets experiencing diarrhea. After 21 days of feeding, we will integrate histological morphology data, intestinal metabolomics, and intestinal proteomics to comprehensively assess the role of YSL in the intestinal health of diarrheic piglets. Our ultimate objective is to determine the feasibility of YSL as a novel feed additive for treating diarrhea in weaned piglets from the perspective of intestinal physiology.

## 2. Materials and Methods

### 2.1. Establishment of a Piglet Diarrhea Model

All experimental procedures were approved by the Institutional Animal Care and Use Committee of Southwest Minzu University, following the guidelines of the Sichuan Council on Animal Care (Ministry of Science and Technology, China, revised in June 2004).

The YSL used in this study was extracted from the abomasal mucosa of yaks, with the detailed method described previously [15]. The abomasal mucosa of yaks was collected and homogenized in ammonium acetate buffer. The supernatant was obtained by centrifugation, and then subjected to pH adjustment (from 4.0 to 5.0), boiling water bath (100 °C, 2 min), and 0.22 μm filtration to prepare the crude extract. A two-step chromatography method was performed on an AKTA protein purification system, using a CM Sepharose FF column (GE Healthcare Life Sciences, Piscataway, NJ, USA) and a Bio-Gel P-100 column (BIO-RAD, Hercules, CA, USA) sequentially. The Bio-Gel P-100 column was equilibrated with 0.2% acetic acid. The solution of YSL was freeze-dried to obtain YSL powder, which was added to the piglet diet at a dosage of 100,000 U/kg.

The experiment used 18 outbred three-way crossbred piglets (Duroc × Large White × Landrace), weaned at 30 days of age, which were obtained from Sichuan Nanxing’ao Agricultural Technology Co., Ltd. (Leshan, China). The piglet toxicity feeding trial was conducted in a fully enclosed facility disinfected with formaldehyde in Ya’an, Sichuan Province. Throughout the feeding trial, the health status of the piglets was monitored daily. The feeding schedule consisted of three daily meals per day (8:00, 12:00, and 18:00), ensuring that a small amount of residual feed remained in the trough before each feeding. Each pen was equipped with nipple-type drinkers, allowing the piglets free access to water. During the trial, pig pen waste was promptly removed to maintain cleanliness, and the environment was kept warm and well-ventilated. The feeding troughs were regularly cleaned to ensure hygiene. The experiment was divided into three groups: the normal control group (Control, C group) with n = 6, the diarrhea group (Diarrhea, D group) with n = 6, and the YSL-treated group (YSL Treatment, YLT group) with n = 6. The number of experimental animals met both statistical and ethical requirements.

**Figure 1:**
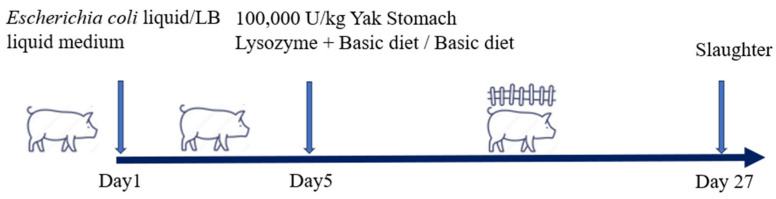
Timetable for YSL treatment in a piglet diarrhea model induced by ETEC.

After the piglets were brought back to the experimental farm, they were kept in a quiet environment for 7 days to acclimatize to the surroundings. Diarrhea in the piglets of the D and YLT groups was induced through continuous oral administration of ETEC K88 culture, while the C group received the same volume of LB liquid culture medium. The ETEC K88 was purchased from Shanghai Xuanya Biotechnology (Shanghai, China), with the catalog number D24607. During this period, all three groups were fed a basal diet. On the fifth day, as all piglets in the D group and YLT group began to exhibit diarrhea, the YLT group commenced feeding a diet supplemented with YSL, while the D and C groups continued with the basic diet. The basal diet for weaned piglets was formulated with reference to the nutrient requirements for pigs specified in NRC (2012). The formula and nutritional levels of the basal diet are shown in Table 1. After maintaining the feeding regimen for 21 days, the subjects were slaughtered. Figure 1 illustrates the YSL treatment timeline in the ETEC-induced piglet diarrhea model.

### 2.2. Determination of Growth Performance in Piglets

During the experimental period, all piglets were weighed weekly while fasting, and their feed intake was recorded daily. After the trial, we calculated the average daily gain (ADG), average daily feed intake (ADFI), and feed-to-gain ratio (F/G) using the following formulas: − ADG = (final weight − initial weight)/number of experimental days − ADFI = total feed consumption across treatments/(number of experimental days × the number of animals in each group) − F/G = ADFI/ADG. Due to group housing in the same pen, average daily feed intake per group was used instead of individual data. Diarrhea scores were recorded based on the severity of the condition, following a scoring system outlined in Table 2 [2]. The diarrhea index was calculated as follows: Diarrhea index = sum of diarrhea scores/(total number of tested piglets × number of experimental days). According to the scoring table, when the diarrhea scores of all piglets in both the YLT group and the D group reached 2 or 3, the piglets in the YLT group were fed with the diet supplemented with YSL, and the experiment officially commenced.

### 2.3. Sample Collection

After the conclusion of the weaned piglet rearing phase, a slaughter experiment was conducted on the piglets. The duodenal contents from each group of piglets were collected and preserved in liquid nitrogen for subsequent determination and analysis of intestinal metabolite dynamics. Aseptic surgical scissors were used to excise segments of the duodenal tissue. One portion was thoroughly rinsed with physiological saline and then immersed in a 4% paraformaldehyde solution for fixation, facilitating the morphological assessment of the intestine. Another portion was aliquoted into cryovials and preserved in liquid nitrogen for TMT-labeling and quantitative proteomic analysis.

### 2.4. Serum Biochemical Indexes Are Measured

Before sacrifice, 20 mL of blood samples were collected from all fasted piglets via the anterior vena cava. The samples were allowed to stand for 30 min and then centrifuged at 4 °C at 3000 rpm for 10 min. The serum from the upper layer of the collection tubes was extracted for the assessment of biochemical and immunological parameters. The concentrations of serum glucose (GLU), urea (UREA), total protein (TP) and albumin (ALB) were analyzed using veterinary automatic biochemical analyzer (Model: BS—240VET, manufactured by Shenzhen Mindray Bio—Medical Electronics Co., Ltd., Shenzhen, China) and the matching commercial detection kits (Shenzhen Mindray Bio-Medical Electronics Co., Ltd., Shenzhen, China).

### 2.5. Hematoxylin and Eosin Staining (H&E Staining)

Fixed in a solution of over 4% formaldehyde, the intestinal tissues were extracted and dehydrated using an automated tissue processor. Subsequently, the specimens were embedded in paraffin wax, followed by a series of procedures that included dehydration, trimming, embedding, sectioning, staining, and mounting. Finally, microscopic examination was conducted. Images of the sections were captured using the BA210 Digital three-eyed microscope system. Initially, all tissues were observed under low magnification to determine the positions for photographic collection, and three random images at 40× magnification were selected. The heights of the intestinal villi and the depths of the crypts were measured, allowing for the calculation of the ratio of villus height to crypt depth (V/C) [17].

### 2.6. Non-Targeted Metabolomics of Duodenal Tissue

Thaw the duodenal tissue, stored in liquid nitrogen, at 4 °C, weighing approximately 30 mg. Add 2 steel beads and incorporate 400 μL of a pre-chilled methanol–water (4:1, *v*/*v*) solution, then homogenize the mixture using a tissue disruptor. After homogenization, thoroughly mix in an additional 600 μL of the pre-chilled methanol–water (4:1, *v*/*v*) solution and incubate the mixture in an ice bath for 20 min. Subsequently, allow the mixture to sit at −20 °C for 1 h, followed by centrifugation at 16,000× *g* for 20 min at 4 °C. Collect the supernatant and evaporate it using a high-speed vacuum concentrator. For mass spectrometry analysis, reconstitute the sample with 100 μL of a methanol–water (1:1, *v*/*v*) solution, centrifuge at 20,000× *g* for 15 min at 4 °C, and analyze the supernatant. The samples are separated using a SHIMADZU-LC30 ultra-high-performance liquid chromatography (UHPLC) system, followed by mass spectrometric analysis with a QE Plus mass spectrometer (Thermo Scientific, Waltham, MA, USA). Finally, the raw data will be processed using MSDIAL (version 5.5) for peak alignment, retention time correction, and peak area extraction, with subsequent pattern recognition conducted using Python software (version 3.10) after pre-processing the data through Unit Variance Scaling (UV) for further data analysis.

### 2.7. Proteomics Analysis

We ground the duodenal samples in liquid nitrogen, added an appropriate volume of SDT lysis buffer, and transferred the mixture to Eppendorf tubes. The samples were then subjected to a boiling water bath for 3 min, followed by ultrasound treatment for 2 min. Subsequently, the samples were centrifuged at 16,000× *g* for 20 min at 4 °C, and the supernatant was collected for protein quantification using the BCA method, where 300 µg of protein from each sample underwent enzymatic digestion. Equal amounts of peptides from each sample were labeled according to the instructions provided in the TMT labeling kit from Thermo Fisher (Waltham, MA, USA). The labeled peptides were mixed in equal proportions and subjected to fractionation using a high-pH reversed-phase chromatography column (Pierce™ High pH Reversed-Phase Peptide Fractionation Kit, Thermo Fisher). Ultimately, the samples were combined into ten distinct fractions. After drying the peptides within each fraction, they were reconstituted in 0.1% formic acid, and an adequate amount was extracted from each sample for chromatographic separation using the Vanquish Neo UHPLC system (operated by the Neo UHPLC chromatography system, Thermo Scientific). The resulting LC-MS/MS raw files were imported into the Proteome Discoverer software (version 3.1, Thermo Scientific) to conduct database searches using the CHIMERYS engine on the Ardia Server. Finally, comprehensive bioinformatics analyses were performed on all protein data, which included identification and quantification analysis, differential expression analysis, and functional analysis. The identified proteins were represented using histograms and Venn diagrams, while differentially expressed proteins were analyzed through volcano plots and clustering analysis. Functional analysis encompassed Gene Ontology (GO) and Kyoto Encyclopedia of Genes and Genomes (KEGG) analyses, enabling the assessment of inter-group differential protein expression and functional relationships.

### 2.8. Statistical Analysis

All data in the experiment were initially sorted using Excel 2019, and then subjected to one-way analysis of variance (ANOVA) with SPSS 20 statistical software. Duncan’s multiple range test was employed for post hoc comparisons. Levene’s test confirmed the homogeneity of variances among groups (*p* > 0.05), and the data conformed to a normal distribution. Therefore, parametric tests were adopted, and non-parametric tests were not required. A *p*-value range of 0.050 < *p* < 0.10 was defined as a significant trend, while *p* < 0.050 indicated statistical significance.

Metabolomics: The raw mass spectrometry data were processed using MS-DIAL for peak alignment, retention time correction, and peak area extraction. All multivariate data analyses and modeling were conducted using R (version 4.0.3) with R packages, applying Pareto scaling for mean data centering. Comparisons between two groups were performed using OPLS-DA, filtering for variables with a VIP score greater than 1 and a *p*-value less than 0.05. For multi-group comparisons, one-way ANOVA was employed to analyze the significance of metabolites across groups, subsequently filtering metabolites with a *p*-value less than 0.05 and an OPLS-DA VIP score greater than 1 as statistically significant. KEGG pathway analysis was conducted using the KEGG database’s differential metabolite data (http://www.kegg.jp) (accessed on 3 November 2025). The KEGG enrichment analysis employed Fisher’s exact test with false discovery rate (FDR) correction for multiple testing. The enriched KEGG pathways were considered statistically significant at a nominal level of *p* < 0.05.

Proteomics: The raw LC-MS/MS files were processed using Proteome Discoverer 2.4 (version 1.6.0.16), followed by bioinformatics data analysis utilizing Perseus software (version 1.5.5.3). Comparisons between two groups employed a Student’s *t*-test combined with fold-change criteria to identify significantly different proteins (fold change ≥ 1.2 and *p*-value < 0.05). Multi-group comparison analysis utilized one-way ANOVA to determine proteins with expression differences, selecting those with *p* < 0.05. To annotate the sequences, information was extracted from UniProtKB/Swiss-Prot, the Kyoto Encyclopedia of Genes and Genomes, and Gene Ontology. Enrichment analyses for GO and KEGG were conducted using Fisher’s exact test, followed by false discovery rate (FDR) correction for multiple comparisons. The enriched GO and KEGG pathways were considered statistically significant at a nominal level of *p* < 0.05.

## 3. Results

### 3.1. Effects of Dietary Supplementation of YSL on the Growth Performance of Weaned Piglets Challenged with ETEC

No significant differences were observed in growth performance metrics among the three groups of piglets (*p* > 0.05). The average daily weight gain and average daily feed intake of piglets in the YLT group were both higher than those in the other two groups; however, these differences did not reach statistical significance (*p* > 0.05) (Table 3). During the trial period from days 5 to 12, the diarrhea index for piglets in the D and YLT groups was significantly higher than that of the C group (*p* < 0.05). From days 12 to 19, significant differences in diarrhea indices were noted among the groups (*p* < 0.05), and from days 19 to 28, D group had a significantly higher diarrhea index than C and YLT groups (*p* < 0.05) (Table 4).

### 3.2. Effects of Dietary Supplementation of YSL on the Intestinal Morphology of Weaned Piglets Challenged with ETEC

**Figure 2:**
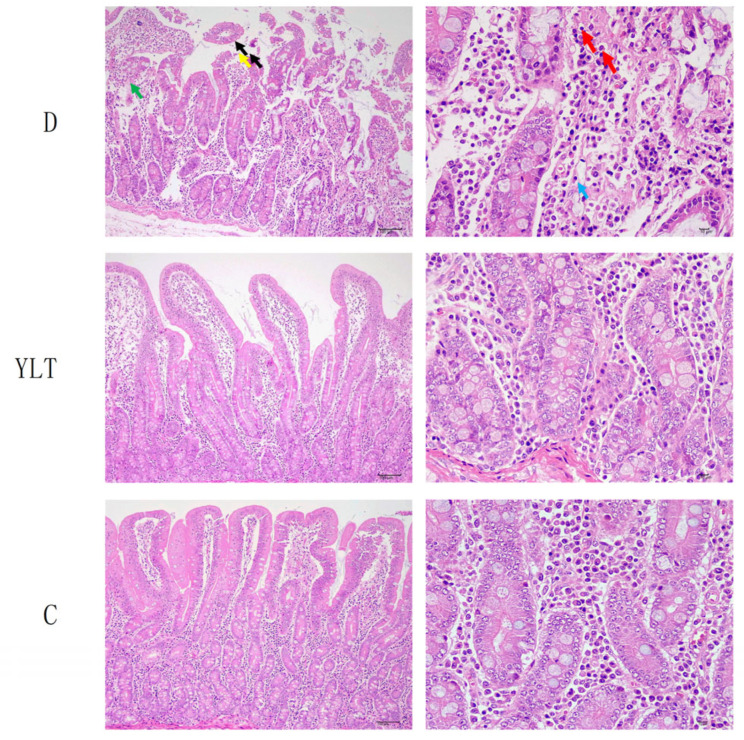
Histopathological observations of the intestines in weaned piglets. The green arrow indicates small intestinal villus shedding; the yellow arrow marks skin cell shedding; a decrease in the number of cells is shown by the black arrows; plasma cells are highlighted with blue arrows; and lymphocytes are indicated by the red arrow. (**Left**) image: 100× magnification; (**right**) image: 400× magnification.

Evaluations of intestinal morphology across three groups revealed that the ETEC treatment significantly affected all measured parameters of intestinal morphology in piglets. As shown in Table 5, compared with the C group, exposure to ETEC in the D and YLT groups significantly increased the duodenal crypt depth (*p* < 0.05) and significantly decreased the V/C (*p* < 0.05). However, there were no significant differences in either crypt depth or the V/C between the D group and the YLT group (*p* > 0.05). In comparison with the D group, the villus height in the YLT group showed a tendency to increase (*p* = 0.085), and there was no significant difference when compared with the C group (*p* > 0.05). Further histological examination via H&E staining of the intestinal tissues (Figure 2) revealed that Group C maintained a more intact structure in the mucosa, submucosa, muscularis, and serosa layers, with well-defined tubular intestinal gland structures and normal morphology of glandular epithelial cells. In contrast, Groups D and YLT exhibited varying degrees of epithelial cell shedding in the intestinal villi, loss of small intestinal villi, and reduced goblet cells; however, no overt inflammatory infiltration was observed in any of the groups.

### 3.3. Effects of Dietary Supplementation of YSL on the Serum Biochemical Indices of Weaned Piglets Challenged with ETEC

**Figure 3:**
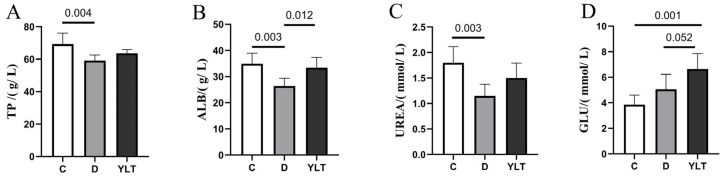
Effect of YSL on serum biochemical indexes of weaned piglets. (**A**) TP; (**B**) ALB; (**C**) UREA; (**D**) GLU. C, D, and YLT represent different groups. The values above the bars indicate the statistical significance (*p*-values) between groups.

The serum biochemical indicators of three groups of piglets are illustrated in Figure 3. The TP level in group D piglets is significantly lower than that in group C (*p* < 0.01). Although the TP level in group D is also lower than that in group YLT, this difference is not statistically significant (*p* > 0.05). The ALB concentration in group D is significantly lower than in group YLT (*p* < 0.05) and markedly lower than in group C (*p* < 0.01). Additionally, the UREA level in the serum of group D piglets is significantly lower than that in group C (*p* < 0.01) and also lower than in group YLT, although this difference is not significant (*p* > 0.05). Conversely, the GLU concentration in the serum of group YLT is significantly higher than that in group C (*p* < 0.01), and there is a trend toward increased serum GLU concentrations when compared to group D (*p* = 0.052).

### 3.4. Effects of Dietary Supplementation of YSL on the Metabolome of Small Intestinal Contents in Weaned Piglets Challenged with ETEC

**Figure 4:**
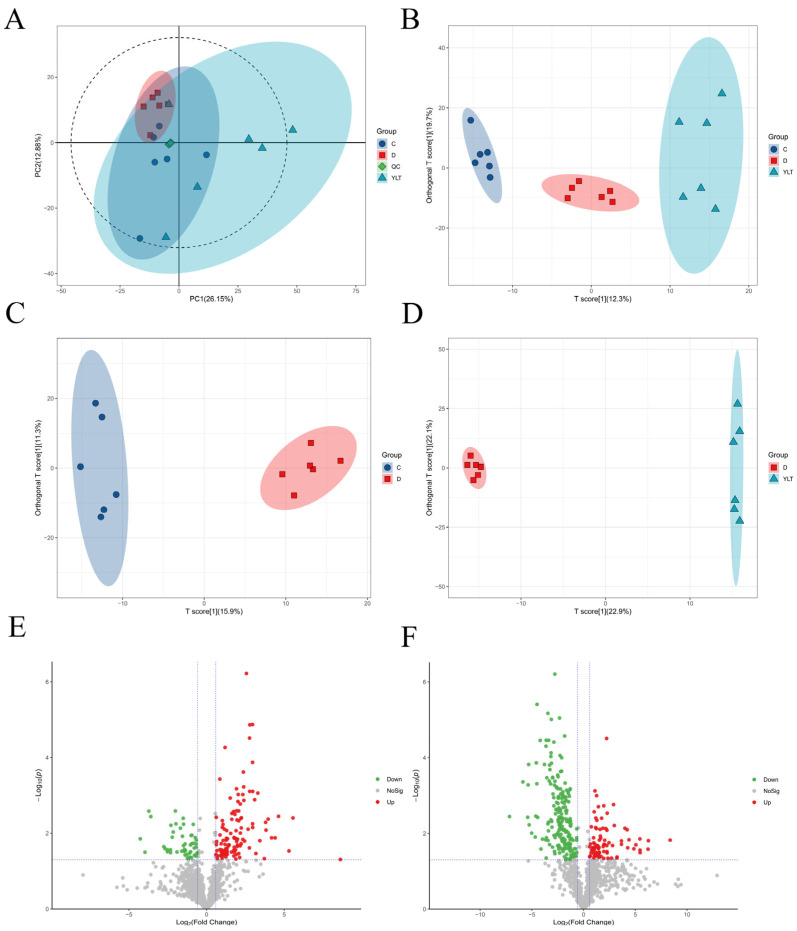
Analysis of differential metabolites among groups. (**A**) PCA of all samples, including QC samples. (**B**–**D**) OPLS-DA, (**B**) global (YLT, C, D); (**C**) C vs. D; (**D**) D vs. YLT. (**E**,**F**) Volcanic map of differential metabolites, (**E**) D vs. C, (**F**) YLT vs. D.

A non-targeted metabolomics analysis was conducted to assess the metabolic alterations following treatment with YSL to evaluate the metabolic profiles of duodenal samples from groups YLT, C, and D (Figure 4). Figure 4A illustrates the principal component analysis (PCA) of all samples, including quality control (QC) samples, which cluster closely together, indicating the reliability of the experimental data. Supervised orthogonal partial least squares discriminant analysis (OPLS-DA) was performed using data from groups D, C, and YLT, revealing an R^2^ intercept of 0.951 and a Q^2^ intercept of −0.16, confirming the robustness and validity of the model (Figure 4B). Furthermore, comparisons between groups were conducted using the OPLS-DA model, yielding significant differences among the groups (Figure 4C,D), with screening criteria set at variable importance in projection (VIP) > 1 and a *p*-value < 0.05. All differential metabolites are visualized through volcano plots (Figure 4E,F). A total of 112 metabolites were significantly upregulated, while 44 were significantly downregulated compared to D vs. C (Figure 4E). In comparing YLT vs. D, 81 metabolites were significantly upregulated, and 179 metabolites were significantly downregulated (Figure 4F). A comparison of the differential metabolites between the two groups revealed that various amino acids and amino acid derivatives (such as leucine acid, GABA, and tryptophan), bile acids (such as chenodeoxycholic acid and ursodeoxycholic acid), vitamins (such as nicotinic acid), and indoles and their derivatives (such as indole-3-lactic acid) were significantly downregulated in D vs. C. At the same time, they were upregulated in YLT vs. D (*p* < 0.01). The complete list of common metabolites and their regulation statuses is provided in Supplementary Table S1.

**Figure 5:**
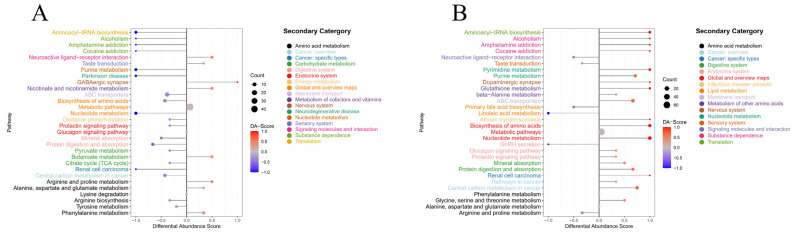
KEGG function annotations of identified proteins between groups, (**A**) D vs. C, (**B**) YLT vs. C.

We conducted a metabolic pathway enrichment analysis of the differential metabolites based on the KEGG database. The top 20 metabolic pathways are illustrated in Figure 5. We observed that the pathways enriched with differential metabolites between the D vs. C and YLT vs. C groups are highly analogous. Notable pathways, such as aminoacyl-tRNA biosynthesis, purine metabolism, ABC transporters, nucleotide metabolism, biosynthesis of amino acids, prolactin signaling pathway, glucagon signaling pathway, mineral absorption, and protein digestion and absorption, exhibited a significant downregulation of differential metabolites in the D vs. C group. In contrast, these metabolites were significantly upregulated in the YLT vs. C group. Additionally, metabolites within pathways such as the citrate cycle (TCA cycle) and tyrosine metabolism were significantly downregulated in the D vs. C group. In contrast, those within the glutathione metabolism, glycine, serine, and threonine metabolism, and beta-alanine metabolism pathways were significantly upregulated in the YLT vs. C group. These pathways and signaling routes are crucial for maintaining intestinal health, regulating immune responses, and promoting nutrient absorption and energy metabolism, indicating that treatment with YSL could ameliorate the intestinal dysfunction caused by ETEC in piglets.

### 3.5. Effects of Dietary Supplementation of YSL on the Small Intestinal Proteome of Weaned Piglets Challenged with ETEC

**Figure 6:**
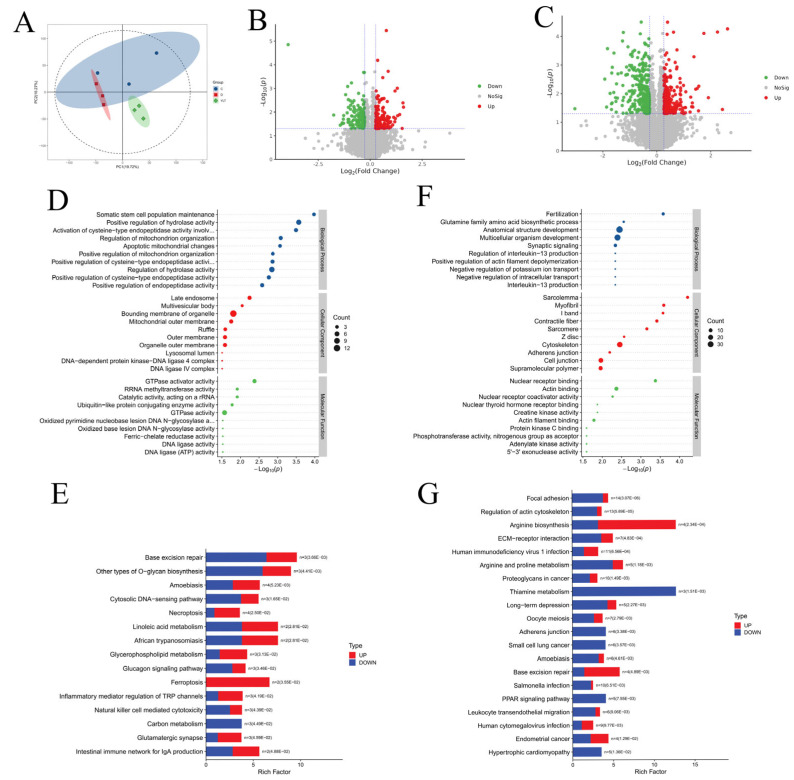
The analysis of identified proteins. (**A**) PCA analysis plot; (**B**,**C**) Volcanic plots of identified proteins between groups: (**B**) D vs. C, (**C**) YLT vs. D; (**D**,**E**) GO enrichment analysis plots of identified proteins between groups: (**D**) D vs. C, (**E**) YLT vs. D; (**F**,**G**) KEGG enrichment analysis plots of identified proteins between groups: (**F**) D vs. C, (**G**) YLT vs. D.

Using the TMT to detect changes in the proteomic profiles of the duodenal mucosa among groups C, D, and YLT, a total of 9313 proteins were identified in the intestines of piglets. PCA revealed distinct clustering patterns among the three groups, indicating that pathogen exposure induced significant alterations in the duodenal proteome of piglets. Notably, the YLT and D groups showed clear separation, suggesting marked proteomic differences between these two groups. Statistical analysis revealed a total of 298 differentially expressed proteins between Group D and Group C, with 154 proteins upregulated and 144 proteins downregulated in Group D compared to Group C. In YLT vs. D, there were 237 proteins upregulated and 334 significantly downregulated, resulting in 571 differentially expressed proteins (Figure 6B,C). A detailed analysis of the common differentially expressed proteins between D versus C and YLT versus D indicated that, following the administration of YSL, 23 significantly downregulated metabolic proteins in the diarrhea group were upregulated, including PHD finger protein 23, Prostaglandin-H2 D-isomerase, and Tight junction-associated protein 1. Conversely, 25 significantly upregulated metabolic proteins, such as C2 domain-containing protein, and Calcium-activated chloride channel regulator 1, were downregulated. These findings suggest that feeding YSL appears to reverse these differential metabolites to varying degrees, indicating that YSL may effectively restore the disrupted intestinal metabolism in diarrhea-affected piglets to levels observed in the normal group (Supplementary Table S2).

To further investigate the pathogenic molecular mechanisms of ETEC and the effect of YSL, a GO functional clustering analysis was conducted on the differentially expressed proteins. The results indicated that the differentially expressed proteins in the D vs. C comparison were primarily localized in structures such as late endosomes, multivesicular bodies, organelle bounding membranes, and mitochondrial outer membranes. These proteins were predominantly involved in various biological processes, including the maintenance of somatic stem cell populations, positive regulation of hydrolase activity, activation of cysteine-type endopeptidase activity, regulation of mitochondrial organization, apoptotic changes in mitochondria, and positive regulation of mitochondrial organization. In terms of molecular functions, these proteins were also associated with GTPase activator activity, Rrna methyltransferase activity, catalytic activity on Rrna, and GTPase activity (Figure 6D).

In the YLT vs. D group, the differentially expressed proteins were predominantly localized in structures such as the sarcolemma, myofibrils, contractile fibers, cytoskeletons, cell junctions, and supramolecular polymers. These proteins were closely associated with biological processes including anatomical structure development, multicellular organism development, and synaptic signaling. Regarding molecular functions, they were linked to nuclear receptor binding, actin binding, actin filament binding, and nuclear thyroid hormone receptor binding (Figure 6E).

The KEGG pathway analysis of differentially expressed metabolites indicated that the proteins in the D vs. C group were significantly enriched in 15 pathways. Notable pathways included base excision repair, various types of O-glycan biosynthesis, amoebiasis, the cytosolic DNA-sensing pathway, necroptosis, linoleic acid metabolism, African trypanosomiasis, glycerophospholipid metabolism, the glucagon signaling pathway, and ferroptosis (Figure 6F). In contrast, the differentially expressed proteins in the YLT vs. D group were significantly enriched in 45 pathways, which included focal adhesion, regulation of the actin cytoskeleton, arginine biosynthesis, ECM–receptor interaction, human immunodeficiency virus type 1 infection, arginine and proline metabolism, proteoglycans in cancer, thiamine metabolism, long-term depression, and oocyte meiosis pathways (Figure 6G).

Interestingly, both the D vs. C and YLT vs. D differential proteins were enriched in the same pathway—base excision repair. Notably, in the D vs. C group, a greater number of differential proteins were downregulated within this pathway, while in the YLT vs. D group, a larger number of differential proteins were upregulated. Additionally, all proteins in the PPAR signaling pathway were downregulated in the YLT vs. D group, suggesting that both the PPAR signaling pathway and base excision repair may play crucial roles in YSL treatment for diarrhea.

### 3.6. Protein–Protein Interaction (PPI) Network Analysis

**Figure 7:**
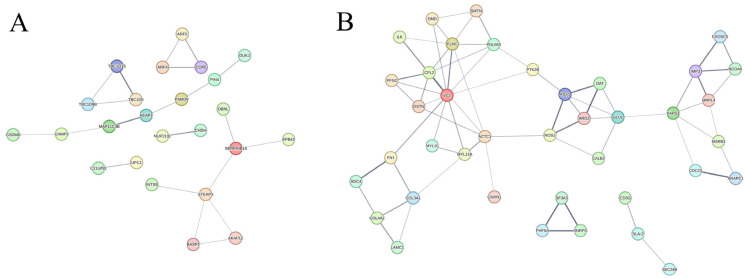
Analysis of PPI networks of differentially expressed proteins. (**A**) D vs. C. (**B**) YLT vs. D. Different colored nodes represent different proteins, and the thickness of the connecting lines between nodes corresponds to different confidence levels, that is, the higher the credibility of the interaction relationship between proteins, the thicker the connecting lines.

The PPI network of differentially expressed proteins enables us to gain a better understanding of the key proteins among various groups. Functional connections between proteins were established using the STRING database (Figure 7). In the D vs. C comparison, proteins SERPINB10 and STEAP3 displayed high connectivity (Figure 7A). In the YLT vs. D comparison, VCL exhibits the highest connectivity, suggesting it may play a crucial role (Figure 7B). Additionally, proteins such as ACTC1, FLNC, CFL2, KARS1, GLUL, ASS1, ARG2, and MRPL4 also exhibited relatively high connectivity. Enrichment analysis was performed on the YLT vs. D associated proteins (Supplementary Figure S1). It was found that these proteins were mainly enriched in the Glutamine family amino acid biosynthetic process, Arginine metabolic process, Glutamine family amino acid metabolic process, Actin filament fragmentation, Muscle structure development, and Actomyosin structure organization.

### 3.7. Integrated Analysis of Metabolome and Proteome

**Figure 8:**
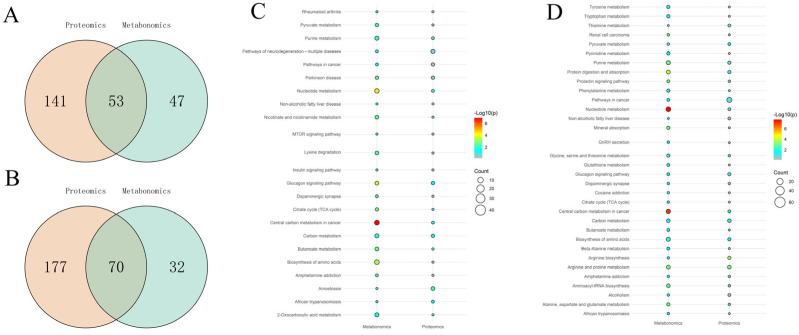
Analysis of common KEGG pathways in metabolome and proteome between groups. (**A**,**B**) Venn diagram of common KEGG pathways between metabolome and proteome (**A**) D vs. C, (**B**) YLT vs. D. (**C**,**D**) Bubble of the common KEGG pathways in metabolome or proteome between groups. (**C**) D vs. C, (**D**) YLT vs. D.

The KEGG pathways shared by metabolomics and proteomics were analyzed (Figure 8). In the D vs. C comparison, there were 53 shared pathways between proteomics and metabolomics (Figure 8A), while in the YLT vs. D comparison, the number of shared pathways was 70 (Figure 8B). In the D vs. C group, Central carbon metabolism in cancer and Nucleotide metabolism were enriched in metabolomics analysis, while Amoebiasis and Carbon metabolism were significantly enriched in proteomics analysis (Figure 8C). In the YLT vs. D group, pathways such as Central carbon metabolism in cancer and Nucleotide metabolism were significantly enriched in both metabolomics and proteomics. ABC transporters and Mineral absorption showed greater significance in metabolomics, whereas Pathways in cancer and Focal adhesion were more prominent in proteomics analysis (Figure 8D).

## 4. Discussion

In this study, we investigated the relationship between the metabolomic profiles of piglets’ intestinal contents and their intestinal tissues’ proteomic profiles under YSL as supplementation. To the best of our knowledge, this is the first research specifically evaluating the impact of YSL on the gut metabolome and proteome of weaned piglets.

Intestinal structural integrity, particularly villi and crypts, is crucial for nutrient absorption [18,19]. In this study, we observed substantial damage to the intestinal structure in the toxin-treated groups (Group D and YLT), characterized by an increased crypt depth and a lower V/C. The villus height in the YLT group tended to be higher than that in the D group (*p* = 0.085), which is similar to that reported previously [20]. Further staining observations revealed that both Groups D and YLT exhibited intestinal villus epithelial shedding and villus loss, with milder epithelial shedding in Group YLT. These findings indicate that the experimental model was successfully established and that dietary supplementation with YSL may improve intestinal morphology in toxin-challenged piglets. Overall, there were no significant differences in growth performance or intestinal indices between the YLT group and D group, except for a trend of difference in villus height (*p* = 0.085). This may be attributed to the spontaneous recovery of physiological functions in piglets of both groups during the 27-day post-ETEC-challenge feeding period, during which the piglets were provided with adequate food, drinking water, and a suitable living environment.

Serum biochemical indicators are essential metrics for assessing the physiological equilibrium within animal organisms, closely linked to metabolic processes involving substances and energy [21]. The levels of TP and ALB in serum can reflect an animal’s ability to digest and absorb dietary protein, and are also used to evaluate nutritional status and liver function [22]. Research has shown that lysozyme increases serum TP and ALB in weaned piglets [23]. Similarly, in this experiment, ALB was also significantly elevated in the YLT group (*p* < 0.05), with no significant difference from the C group (*p* > 0.05). Urea is the final metabolic product of protein nitrogen balance [24]. In this study, the serum UREA and TP content in group C was significantly higher than that in group D (*p* < 0.05), indicating that ETEC challenge reduced the metabolic level in piglets. GLU directly reflects the body’s energy metabolism [25]. GLU absorption stimulates the release of insulin and promotes protein synthesis in animals [26]. Additionally, GLU plays a crucial role in the intestinal development of piglets and nutrient digestion and absorption [27,28]. Our results show that the serum GLU in the YLT group was significantly higher than that in the C group (*p* < 0.05), and tended to increase compared with the D group (*p* = 0.052). These results suggest that YSL can improve metabolism in piglets, enhance their resistance to external adverse stimuli, and thereby strengthen the body’s overall immune response.

Intestinal metabolites influence intestinal health, and their balance is crucial for maintaining intestinal well-being [29,30]. Metabolomic analysis shows that several essential substances for maintaining gut health, such as bile acids (BAs), vitamins, indole, and its derivatives, are significantly upregulated or downregulated in the D vs. C comparison, with the opposite trend in YLT vs. D. BAs regulate energy metabolism by binding to and activating specific receptors [30]. Chenodeoxycholic acid (CDCA) and ursodeoxycholic acid (UDCA) levels in piglets were significantly downregulated after ETEC treatment, and YSL intervention effectively reversed this trend. Studies have shown that UDCA can combat diarrhea in calves caused by ETEC infection [31]. The levels of nicotinic acid, indole, and their derivatives are significantly higher in the YLT group compared to the D group. Nicotinic acid is a precursor for synthesizing coenzymes NAD and NADPnicotinic acid [32], both essential for the redox reactions involved in energy metabolism [33,34]. Metabolites like indoles and phenolics activate the aryl hydrocarbon receptor, which mediates fatty acid metabolism via NAD-dependent pathways [35]. The serum GLU level in the YLT group was significantly increased, providing more substrates for acetyl-CoA production [36]. Subsequently, fatty acid synthesis was driven by NADPH, ultimately promoting fatty acid deposition in adipose tissue and enhancing the animals’ resistance to external stimuli [37,38]. In conclusion, YSL may alleviate the effects of diarrhea on piglets by regulating energy-related metabolic pathways.

The KEGG enrichment results indicate that enzymes associated with purine metabolism, pyrimidine metabolism, nucleotide metabolism, and amino acid biosynthesis are significantly upregulated in the comparison between YLT and D. This finding aligns with previously reported results [39], suggesting that lysozyme may shift gut microbiota towards enhanced amino acid and nucleotide metabolism. Weaning stress and ETEC infection impair piglets’ intestinal barrier function, and supplementing with nucleotides improves gut morphology and reduces the incidence of diarrhea [40]. Interestingly, we found that multiple amino acids were upregulated in the Mineral absorption and Protein digestion and absorption pathways, including tryptophan and serine. Minerals support animal health and production [41]. Amino acid metabolism is crucial for maintaining proper intestinal integrity and alleviating inflammation [42,43], while dietary serine supplementation can reduce the diarrhea rate in piglets [44,45]. Diarrhea can lead to the loss of essential nutrients such as amino acids in the body, and the organism needs to upregulate their biosynthetic pathways to repair damaged tissues. A significant increase in serum GLU levels is conducive to stimulating insulin secretion, promoting protein synthesis and deposition, and ultimately facilitating the repair of intestinal epithelium. Combined with intestinal morphological and serum biochemical results, YSL may promote intestinal amino acid metabolism, thus enhancing nutrient digestion and absorption in the intestine. Consequently, this leads to improved intestinal morphology and barrier integrity in piglets.

The proteomics results indicate that the Base Excision Repair (BER) pathway is significantly enriched in both D vs. C and YLT vs. D comparisons. YSL treatment reverses the downregulation of the POLE3 and APTX proteins within the BER pathway. POLE3 is critical in DNA repair within the long-patch BER enzyme complex [46]. APTX is involved in the repair of DNA single-strand breaks [47,48]. Shiga toxins secreted by ETEC induce DNA breaks and apoptosis in infected cells, and also promote proteolysis in neighboring cells, thereby enhancing host toxicity [49]. In the YLT group, the expression levels of POLE3 and APTX were significantly higher than those in the D group, suggesting that treatment with bovine lysozyme can partially reverse the genetic damage caused by ETEC. The enrichment of the nucleotide synthesis pathway observed in the metabolome corroborates this view.

The differentially expressed proteins involved in the PPAR signaling pathway were downregulated after YSL treatment, including ILK and APOC3. ILK, a serine-threonine protein kinase, enhances pro-inflammatory signaling during immune responses to various stimuli, exacerbating inflammatory reactions [50]. APOC3 is a small apolipoprotein [51]. Lower APOC3 expression is associated with reduced expression of inflammatory factors and alleviation of tissue damage [52]. These results suggest that YSL may alleviate inflammation by downregulating pro-inflammatory pathways.

PPI analysis revealed that VCL exhibited high connectivity with various proteins in the comparison of YLT vs. D. As a ubiquitously expressed cytoskeletal protein localized on the cell membrane, VCL is responsible for maintaining the integrity of intercellular junctions and barrier function in epithelial cells [53]. Additionally, proteins such as ACTC1, FLNC, CFL2, PDLIM3, COL3A1, and COL4A2 also showed high connectivity, which are involved in cytoskeletal construction, regulation of contractile function, and extracellular matrix formation in intestinal epithelial cells [54,55,56,57]. Furthermore, GLUL, ASS1, and ARG2 also exhibit high connectivity, and were enriched in the arginine biosynthesis pathway, and collectively maintain intracellular arginine homeostasis [58,59,60]. In newborn mammals, the intestinal epithelial cells of the small intestine are primarily responsible for the endogenous synthesis of arginine [61], but synthesis may be insufficient during intestinal inflammation or metabolic impairment [62]. Studies have shown that dietary arginine supplementation can enhance intestinal integrity [63], regulating immune responses [64]. Based on PPI analysis, YSL may aid intestinal barrier restoration and diarrhea alleviation by regulating intestinal barrier-related proteins and promoting arginine synthesis. However, the specific roles of these proteins in YSL-mediated intestinal barrier restoration need further verification.

In the integrated analysis, for the YLT vs. D comparison, pathways including Nucleotide metabolism were significantly enriched in both metabolomics and proteomics. This is consistent with the previous discussion on metabolomics, once again indicating that YSL may affect intestinal function by influencing nucleic acid metabolism. Meanwhile, pathways like ABC transporters and Mineral absorption in metabolomics are all related to substance absorption and transport [65,66], suggesting that YSL can promote the absorption of nutrients.

In conclusion, dietary supplementation with YSL may alleviate ETEC-induced diarrhea in weaned piglets through a dual-pathway mechanism involving “energy metabolism regulation and intestinal repair support”. On one hand, YSL positively regulates the levels of intestinal BAs, indole derivatives, and nicotinic acid, which optimizes energy metabolism. This regulatory effect is reflected by the significantly increased serum GLU levels in the YLT group, providing energy for the piglets to resist ETEC-induced damage. On the other hand, YSL promotes nucleotide metabolism in the small intestinal epithelium and the expression of functional proteins, such as GLUL and ASS1 as well as VCL and ACTC1. These changes facilitate intestinal mucosal repair and nutrient absorption, thereby laying a material foundation for intestinal function recovery.

Notably, the present study only identified potential molecules and pathways involved in YSL-mediated diarrhea alleviation via multi-omics analysis, without verifying the direct regulatory effect of these differential molecules on diarrhea phenotypes. In future research, targeted experiments focusing on key metabolites and proteins should be designed to provide more rigorous mechanistic support for YSL as a potential antibiotic alternative.

## 5. Conclusions

Overall, due to the unique properties of YSL, this study demonstrates that dietary supplementation with YSL exerts an effect against diarrhea induced by ETEC. It regulates energy metabolism to support the body’s damage resistance, promotes nucleotide and functional protein production in intestinal epithelial cells, and these multi-pathway effects synergistically counteract ETEC-induced intestinal damage, ultimately alleviating morphological impairment and enhancing barrier function. This work confirms YSL’s potential as a green alternative to antibiotics for treating weaned piglet diarrhea, laying a theoretical and experimental foundation for its application in sustainable livestock production.

## Figures and Tables

**Table 1:** Composition and nutrient levels of basal diets (air-dry basis, %).

Ingredient	Content (%)
Corn	32.56
Bran	4.31
Soybean meal	15.82
Whey powder	4.94
Calcium powder	0.69
Dicalcium phosphate	0.40
Soybean protein	4.94
Wheat	29.66
Lys (98%)	0.56
Met	0.16
Thr	0.20
Soybean oil	1.48
Oligosaccharide	3.96
Premix ^(1)^	0.3
Total	100
Nutrient composition ^(2)^	
Digestible Energy (MJ/kg)	14.51
Crude protein	18.37
Calcium	0.81
Total phosphorus	0.59
Digestible phosphorus	0.42

^(1)^ The premix provides the following components per kilogram of the basal diet: vitamin A 12,000 IU, vitamin B_1_ 2.5 mg, vitamin B_2_ 4 mg, vitamin B_6_ 7 mg, vitamin D_3_ 2000 IU, vitamin E 30 IU, vitamin K_3_ 2.5 mg, biotin 80 μg, folate 40 mg, D-pantothenate acid 12.5 mg, nicotinic acid 40 mg, Fe 100 mg, Cu 8 mg, Zn 100 mg, Mn 20 mg, I 0.36 mg, Se 0.48 mg. ^(2)^ All nutritional levels are calculated values based on the formulation of the basal diet.

**Table 2:** The diarrhea evaluation of piglets.

Level of Diarrhea	Fecal Appearance	Diarrhea Score
Normal	Formed or granular	0
Mild diarrhea	Moist or soft feces	1
Moderate diarrhea	Thick liquid feces or mild diarrhea	2
Severe diarrhea	Watery feces and severe diarrhea	3

**Table 3:** Effect of YSL on the growth performance of weaned piglets infected with ETEC.

Items	C	D	YLT
BW, kg	27.60 ± 3.05	26.05 ± 4.54	29.00 ± 5.26
ADG, kg	0.59 ± 0.06	0.54 ± 0.13	0.61 ± 0.14
ADFI, kg	0.85	0.8	1.05
F/G	1.47 ± 0.16	1.55 ± 0.40	1.74 ± 0.29

Note: No significant differences were observed among groups (*p* > 0.05), so no superscripts are used to indicate differences in this table.

**Table 4:** Effect of YSL on the diarrhea index of weaned piglets infected with ETEC.

Items	Group		
	C	D	YLT
Days 5 to 12	0.19 ± 0.22 ^b^	1.71 ± 0.36 ^a^	1.57 ± 0.22 ^a^
Days 12 to 19	0.14 ± 0.13 ^c^	1.00 ± 0.29 ^a^	0.59 ± 0.17 ^b^
Days 19 to 28	0.14 ± 0.13 ^b^	0.57 ± 0.40 ^a^	0.19 ± 0.12 ^b^

Note: Peers labeled with different letters indicate significant differences (*p* < 0.05), while those labeled with the same letter indicate non-significant differences (*p* > 0.05).

**Table 5:** Effect of YSL on the intestinal morphology of piglets infected with ETEC.

Group	C	D	YLT
Duodenum			
Villi height/μm	639.59 ± 89.12 ^a^	519.70 ± 106.40 ^b^	613.31 ± 62.89 ^ab^
Crypt depth/μm	280.57 ± 52.69 ^b^	459.67 ± 82.644 ^a^	523.04 ± 120.12 ^a^
V/C	2.36 ± 0.62 ^a^	1.17 ± 0.34 ^b^	1.21 ± 0.21 ^b^

Note: Peers labeled with different letters indicate significant differences (*p* < 0.05), while those labeled with the same letter indicate non-significant differences (*p* > 0.05).

## Data Availability

The raw metabolomic data in this study have been deposited in MetaboLights with the project accession number [MTBLS13088], while the raw proteomic data have been uploaded to iProX under the project accession number [IPX0013523000]. Rest data will be made available on request.

## Supplementary Materials

The following supporting information can be downloaded at: https://www.mdpi.com/article/10.3390/ani15223287/s1. Table S1: Significant Differential Metabolites Common to D vs. C and YLT vs. D. Table S2: Significant differences in common proteins between D vs. C and YLT vs. D. Figure S1: GO biological process enrichment analysis of PPI - associated proteins in YLT vs D.
