# Supplementary material for: Dietary Supplementation with Yak Stomach Lysozyme Improves Intestinal Health and Nutrient Metabolism in Weaned Piglets Challenged with Enterotoxigenic Escherichia coli (ETEC)
Source: Animals (Basel). 2025 Nov 13;15(22):3287. doi: 10.3390/ani15223287 (PMC12649399; doi:10.3390/ani15223287)

Supplementary Materials

Supplementary Table S1

Significant Differential Metabolites Common to D vs. C and YLT vs. D

Metabolite name	P.value	D vs. C	YLT vs. D
5-Oxooctadecanoic acid	6.20776E-07	DOWN	UP
9,10-Dihydroxystearic acid	3.91063E-06	DOWN	UP
8,12-Octadecadienoic acid	6.74121E-06	DOWN	UP
6,10,14-Trimethyl-5,9,13-pentadecatrien-2-one	9.74574E-06	DOWN	UP
2,3-Dihydrobenzofuran	3.50453E-05	DOWN	UP
2-Methylbenzaldehyde	3.48139E-05	DOWN	UP
Tropic acid	3.46434E-05	DOWN	UP
1,3,5-Hexatriene	3.88404E-05	DOWN	UP
1,4-Dihydro-3H-2-benzopyran-3-one	4.97587E-05	DOWN	UP
Koninginin E	7.47981E-05	DOWN	UP
Ursodeoxycholic acid	9.33838E-05	DOWN	UP
2-Hydroxy-3-(2-hydroxyphenyl)propanoic acid	8.79327E-05	DOWN	UP
7-Hydroxy-5-methylflavone	0.000137021	DOWN	UP
Indole-3-Lactic Acid	0.000157472	DOWN	UP
Leucinic acid	0.000151489	DOWN	UP
GABA	0.000228038	DOWN	UP
Isoalantolactone	0.000341363	DOWN	UP
Betazole	0.000340182	DOWN	UP
Chenodeoxycholylphenylalanine	0.000437294	DOWN	UP
Methylpyrazine	0.000412644	DOWN	UP
Chenodeoxycholylleucine	0.000523433	DOWN	UP
Mannitol	0.00046872	DOWN	UP
7alpha-hydroxy-3-oxochol-4-en-24-oic Acid	0.000493855	DOWN	UP
Tyramine	0.000489279	DOWN	UP
Octadeca-9,12-dienal	0.000796625	DOWN	UP
Pinocarvyl acetate	0.000911084	DOWN	UP
Nicotinic acid	0.001128766	DOWN	UP
cis-15-Octadecenoic acid	0.001198329	DOWN	UP
3-Hydroxy-cis-5-tetradecenoylcarnitine	0.001136664	DOWN	UP
Phenylacetic acid	0.001367961	DOWN	UP
Prolylphenylalanine	0.001466859	DOWN	UP
Acetylglycine	0.00243979	DOWN	UP
2-Hydroxy-2-methylbutyric acid	0.002077344	DOWN	UP
Ethyl (S)-3-hydroxybutyrate glucoside	0.003041686	DOWN	UP
1,2,3,4,Tetrahydro-1,5,7-trimethylnapthalene	0.003901899	DOWN	UP
Indane	0.004035874	DOWN	UP
Coprocholic acid	0.003779998	DOWN	UP
2-Dehydro-O-desmethylangolensin	0.003759634	DOWN	UP
8-Hydroxy-9,10-epoxystearic acid	0.003509903	DOWN	UP

2(1H)-Naphthalenone, 8-[5-(acetyloxy)-3-methylpentyl]octahydro-4,4,8a-trimethyl-7-methylene-	0.004928301	DOWN	UP
Myriocin-12-en	0.005070062	DOWN	UP
3-Hydroxyoctadecanoic Acid	0.004618483	DOWN	UP
Chenodeoxycholic acid	0.005370635	DOWN	UP
Chenodeoxycholylglutamic acid	0.005610568	DOWN	UP
Ophthalmic acid	0.006848391	DOWN	UP
Nootkatone	0.006047698	DOWN	UP
Naringenin	0.005958355	DOWN	UP
3-Phenyllactic acid	0.007149291	DOWN	UP
Chenodeoxycholic acid 3-glucuronide	0.008580414	DOWN	UP
2-Hydroxyisobutyric acid	0.009214947	DOWN	UP
1-(4-hydroxyphenyl)-3-[(2R,3R,4S,5S,6R)-3,4,5-trihydroxy-6-(hydroxymethyl)oxan-2-yl]oxypropan-1-one	0.01806895	DOWN	UP
Phe-CA	0.01126885	DOWN	UP
Tryptophyl-Tryptophan	0.011997966	DOWN	UP
Floionolic acid	0.012500233	DOWN	UP
Tryptamine	0.014831999	DOWN	UP
2-Hydroxyglutaric acid	0.014574006	DOWN	UP
8-Methylquinoline	0.015416847	DOWN	UP
Chenodeoxycholylhistidine	0.023310355	DOWN	UP
Cholyllysine	0.022714262	DOWN	UP
Tricarballylic acid	0.025917153	DOWN	UP
Murideoxycholic acid	0.024550244	DOWN	UP
Chenodeoxycholyllysine	0.029923694	DOWN	UP
Glucose	0.033128048	DOWN	UP
Gln-CDCA	0.041509598	DOWN	UP
Dibutyl phthalate	0.000753317	UP	DOWN
Imidazole-4-acetaldehyde	0.001008617	UP	DOWN
Dehydroascorbic acid	0.001737975	UP	DOWN
DOPA	0.001871651	UP	DOWN
Tryptophan	0.002698456	UP	DOWN
Octopamine	0.004517209	UP	DOWN
Malic acid	0.006208043	UP	DOWN
1H-Imidazole-4-carboxamide	0.007335759	UP	DOWN
2-Cyanoacetamide	0.007031169	UP	DOWN
Cytidine	0.008023777	UP	DOWN
Phthalic anhydride	0.008928143	UP	DOWN
Inosine	0.01399402	UP	DOWN
Hypoxanthine	0.014770689	UP	DOWN
Fumaric acid	0.015814423	UP	DOWN
2-[2-[(Z)-pent-2-enyl]-3-[3,4,5-trihydroxy-6-(hydroxymethyl)oxan-2-yl]oxycyclopentyl]acetic acid	0.017697405	UP	DOWN
Guanine	0.019437742	UP	DOWN
Galactaric acid	0.021512984	UP	DOWN
3,7-Bisaboladiene-2,8-dione	0.024037857	UP	DOWN

Guanosine	0.026100652	UP	DOWN
Ketopinic Acid	0.021985151	UP	DOWN
Xanthosine	0.025580336	UP	DOWN
3-Azetidinecarboxylic acid	0.027133718	UP	DOWN
Benzofuran	0.029432507	UP	DOWN
Tyrosine	0.036898014	UP	DOWN
Dodecanamide	0.04566173	UP	DOWN

Supplementary Table S2

significant differences in common proteins between D vs. C and YLT vs. D.

Gene Symbol/Accession	Protein Name	P.value	D vs. C	YLT vs. D
NAB2	NGFI-A binding protein 2	0.019336173	Down	Down
A0A8D0JTG9	NmrA-like family domain-containing protein 1	0.000591118	Down	Up
TBC1D5	TBC1 domain family member 5	0.001031882	Down	Up
EPB42	Erythrocyte membrane protein band 4.2	0.001515237	Down	Up
PHF23	PHD finger protein 23	0.002924132	Down	Up
A0A8D0VRG0	Prostaglandin-H2 D-isomerase	0.005934733	Down	Up
A0A8D1DZU2	Sodium/myo-inositol cotransporter	0.007034323	Down	Up
UFC1	Ubiquitin-fold modifier-conjugating enzyme 1	0.007662195	Down	Up
A0A8D0PGW0	BAT2 N-terminal domain-containing protein	0.01596853	Down	Up
TJAP1	Tight junction associated protein 1	0.01873318	Down	Up
SLC25A46	Solute carrier family 25 member 46	0.012597493	Down	Up
SMUG1	Single-strand-selective monofunctional uracil-DNA glycosylase 1	0.017925732	Down	Up
CYBC1	Essential for reactive oxygen species protein	0.019713695	Down	Up
MED8	Mediator of RNA polymerase II transcription subunit 8	0.020148046	Down	Up
A0A8D0X2E3	Rho GTPase activating protein 9	0.021994322	Down	Up
SGIP1	SH3-containing GRB2-like protein 3-interacting protein 1	0.027642861	Down	Up
F1S1S8	Uncharacterized protein	0.028289897	Down	Up
NAA16	N(alpha)-acetyltransferase 16, NatA auxiliary subunit	0.030206378	Down	Up
HS2ST1	Heparan sulfate 2-O-sulfotransferase 1	0.033920079	Down	Up
A0A8D0QGC3	C2 domain-containing protein	0.03464966	Down	Up
A0A8D1R4H2	Ran-binding protein 6	0.036969157	Down	Up
SNRPG	Small nuclear ribonucleoprotein G	0.037430171	Down	Up
A0A8D0J3F5	DNA-directed RNA polymerase I subunit RPA34	0.049592222	Down	Up
A0A8D0R070	Small cell adhesion glycoprotein	7.93486E-05	Up	Down
ACAT2	Sterol O-acyltransferase 2	0.001271658	Up	Down
A0A8D0NB67	Hydroxymethylglutaryl-CoA synthase	0.001354477	Up	Down
A0A8D1P9K6	Calcium-activated chloride channel regulator 1	0.002214963	Up	Down
POLE3	DNA polymerase epsilon subunit 3	0.002896907	Up	Down
PPP3R1	Protein phosphatase 3 regulatory subunit B, alpha	0.008118173	Up	Down

SELENBP1	Methanethiol oxidase	0.013128353	Up	Down
KLF13	Kruppel-like factor 13	0.01339448	Up	Down
A0A8D1AG25	threonine--tRNA ligase	0.01380675	Up	Down
TUT1	Speckle targeted PIP5K1A-regulated poly(A) polymerase	0.020510833	Up	Down
PTMA	Prothymosin alpha	0.021641859	Up	Down
C2CD3	C2 domain containing 3 centriole elongation regulator	0.022073256	Up	Down
LOC100513868	Butyrophilin-like protein 1	0.022164188	Up	Down
A0A8D1FLL3	C2 domain-containing protein	0.024720447	Up	Down
A0A8D1GRG1	Peptidase S1 domain-containing protein	0.025684299	Up	Down
STK17B	Serine/threonine kinase 17b	0.025783645	Up	Down
A0A480Z8U9	Guanine nucleotide exchange factor VAV2 isoform 2 (Fragment)	0.033681166	Up	Down
A0A8D0J4V1	Squalene monooxygenase	0.035446634	Up	Down
A0A8D1VPK1	SS18 N-terminal domain-containing protein	0.035804895	Up	Down
REX1BD	Required for excision 1-B domain containing	0.036035511	Up	Down
A0A8D1D323	Transcription factor BTF3	0.040135472	Up	Down
A0A8D0Z187	Trimethyllysine dioxygenase, mitochondrial	0.04017069	Up	Down
TTPAL	Alpha tocopherol transfer protein like	0.047582087	Up	Down
A0A8D1BHT6	L1 transposable element RRM domain-containing protein	0.049478577	Up	Down
A0A480DPB9	Gigaxonin	0.049932195	Up	Down

Supplementary Figure S1

GO biological process enrichment analysis of PPI - associated proteins in YLT vs D

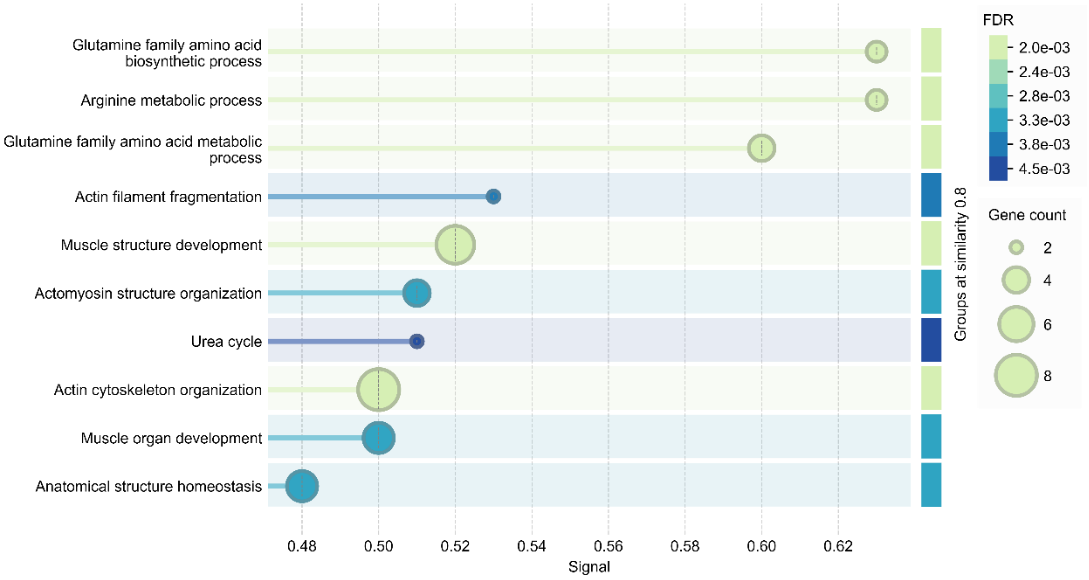

Supplement: Supplementary file 1 [file animals-15-03287-s001.zip › animals-3915310-supplementary.pdf]
